# Role of BDNF in form-deprivation myopia progression in guinea pigs

**DOI:** 10.3389/fimmu.2026.1733238

**Published:** 2026-05-29

**Authors:** Ruo-Yi Xie, Qi Wang, Han Liu, Rui Luo, Chun-Yi Liu, Shi Li, Yong Chai, Fen Xiong

**Affiliations:** 1 Department of Ophthalmology, Jiangxi Provincial Children’s Hospital, Nanchang, Jiangxi, China; 2 Department of Ophthalmology, Jiangxi Maternal and Child Health Hospital, Nanchang, Jiangxi, China; 3 Department of Ophthalmology, Nanchang University Affiliated Eye Hospital, Nanchang, Jiangxi, China; 4 Department of Ophthalmology, Shanghai General Hospital, Shanghai Jiao Tong University School of Medicine, National Clinical Research Center for Eye Diseases, Shanghai Key Laboratory of Ocular Fundus Diseases, Shanghai, China; 5 Shanghai Engineering Center for Visual Science and Photomedicine, Shanghai, China; 6 Shanghai Engineering Center for Precise Diagnosis and Treatment of Eye Diseases, Shanghai, China

**Keywords:** BDNF, form deprivation, guinea pig, intravitreal injection, myopia

## Abstract

**Purpose:**

This study investigated the effects of intravitreal administration of brain-derived neurotrophic factor (BDNF) on the progression of form-deprivation myopia (FDM) in a guinea pig model and explored the associated molecular mechanisms.

**Methods:**

In Experiment 1, 45 pigmented guinea pigs (aged 3 weeks) were randomly assigned to 5 groups to assess the impact of varying BDNF concentrations (50, 100, and 200 μg/mL) delivered via intravitreal injection following 4 weeks of monocular form deprivation. Axial length and refractive error were measured at baseline, after the deprivation period, and one day post-injection. In Experiment 2, 36 guinea pigs were allocated into 4 groups to further assess ocular structural changes and underlying molecular pathways. Hematoxylin and eosin (H&E) staining, immunofluorescence, optical coherence tomography (OCT), and western blotting were used to analyze morphological changes in the retina, choroid, and sclera, as well as the expression of BDNF, phosphoinositide 3-kinase (PI3K), protein kinase B (AKT), endothelial nitric oxide synthase (eNOS), and neuronal nitric oxide synthase (nNOS) in the retina.

**Results:**

The 200 μg/mL BDNF concentration significantly inhibited axial elongation and myopic refractive shifts in eyes with FDM. Immunofluorescence localized BDNF expression predominantly to the retina and choroid. Both H&E staining and OCT imaging demonstrated increased retinal and choroidal thickness and improved scleral collagen organization following BDNF administration. Western blot analysis revealed a downregulation of PI3K, AKT, eNOS, and nNOS expression in the retina of FDM-affected eyes treated with BDNF.

**Conclusion:**

Intravitreal injection of 200 μg/mL BDNF effectively attenuated the progression of FDM in guinea pigs. This effect may be mediated through modulation of the PI3K/AKT/eNOS/nNOS signaling pathway. These findings support BDNF as a potential therapeutic target for myopia control.

## 1 Introduction

Myopia, a common refractive error, has emerged as a major global public health concern due to its rapidly rising prevalence. Epidemiological projections estimate that by 2050, approximately 4.758 billion individuals, accounting for 49.8% of the global population will be affected by myopia, with 938 million individuals (9.8%) developing high myopia (1). High myopia is associated with an increased risk of serious ocular complications, including cataracts, glaucoma, chorioretinal degeneration, and retinal detachment, which can result in irreversible vision loss or blindness (2). The pathogenesis of myopia involves complex interactions between environmental and genetic factors, and despite substantial research efforts, its underlying mechanisms remain incompletely understood (3). Experimental models of form-deprivation myopia (FDM), particularly in guinea pigs, have provided valuable insights into the development of myopia and the associated structural changes in ocular tissues (4).

Brain-derived neurotrophic factor (BDNF), a key member of the neurotrophin family, is predominantly synthesized in retinal ganglion cells (RGCs) and amacrine cells (5). BDNF exerts neurotrophic effects on retinal photoreceptors and RGCs. Previous studies have shown that exogenous BDNF modulates visual cortical plasticity by altering neural electrical activity in response to monocular deprivation (6). Furthermore, intravitreal administration of BDNF in rat models upregulates basic fibroblast growth factor, thereby inhibiting swelling in Müller glial and bipolar cells, preserving retinal thickness, and providing neuroprotective effects (7, 8).

Emerging research highlights the dual role of BDNF in the context of myopia. It functions not only as a neuroprotective agent within the retina but also as a regulatory factor potentially involved in structural remodeling of the eye during myopic progression (9, 10). Although the protective effects of BDNF on the retina and optic nerve have been well documented (11–13), significantly reduced BDNF expression has been observed in the aqueous humor of individuals with high myopia, suggesting a potential association with pathological changes (14–16). A recent study proposed BDNF as a novel therapeutic target for the management of myopia (17). However, most existing studies have focused on changes in BDNF expression resulting from myopia, rather than exploring whether exogenous BDNF supplementation could attenuate disease progression. This study addresses this knowledge gap by evaluating the protective effects of intravitreal BDNF administration in a guinea pig model of FDM.

BDNF exerts its neuroprotective effects primarily through binding to the tropomyosin receptor kinase B (TrkB), which activates three major downstream signaling cascades: the mitogen-activated protein kinase (MAPK) pathway, the phospholipase C-γ (PLCγ) pathway, and the phosphatidylinositol 3-kinase (PI3K)/protein kinase B (AKT) pathway (18). Within the molecular pathways implicated in myopia, the PI3K/AKT signaling pathway has attracted considerable interest due to its regulatory role in cell growth, proliferation, and metabolism (19). Activation of the PI3K/AKT pathway has been associated with increased retinal fibrosis, decreased retinal thickness, and impaired physiological function, all of which contribute to the progression of myopia (20). Nitric oxide (NO), a key mediator of oxidative stress, may exacerbate myopic changes through activation of the PI3K/AKT pathway (21). NO is synthesized by endothelial nitric oxide synthase (eNOS) and neuronal nitric oxide synthase (nNOS), and upregulation of eNOS and nNOS has been reported in the retinas of guinea pigs with FDM (22, 23). A combination of histological, molecular, and imaging techniques was used to assess structural changes in the retina, choroid, and sclera. Changes in the expression levels of PI3K, AKT, eNOS, and nNOS proteins were analyzed to elucidate the molecular mechanisms underlying the therapeutic potential of BDNF. These findings may offer theoretical support for BDNF as a potential therapeutic target for myopia control.

## 2 Materials and methods

### 2.1 Animals and ethics statement

Healthy three-week-old tricolor guinea pigs (Cavia porcellus) of either sex (body weight: 150–200 g) were obtained from Danyang Changyi Experimental Animal Breeding Co., Ltd. (Production License No.: SCXK(Su)2021-0002). Both male and female guinea pigs were used in this study, with approximately equal distribution across experimental groups. All animals were specific pathogen-free (SPF), not genetically modified, and of wild-type genotype. Following a one-week acclimatization period under standard housing conditions (12-h light/dark cycle, 22–24 °C, 50–60% humidity), ophthalmic screening was performed to exclude animals with congenital myopia, keratitis, conjunctivitis, or other ocular abnormalities. Only guinea pigs with interocular refractive error differences ≤ 2.00 diopters (D) were included in the study. No animal had undergone any prior surgical or experimental procedures. Guinea pigs were housed under controlled environmental conditions (25 ± °C; 12-hour light/12-hour dark cycle) with unrestricted access to food and water. Ethical approval for this study was obtained from the Ethics Committee of the Affiliated Eye Hospital of Nanchang University (Approval No.: YLP20250042). All experimental procedures were conducted in accordance with the Association for Research in Vision and Ophthalmology Statement for the Use of Animals in Ophthalmic and Vision Research. At the conclusion of the experiment, guinea pigs were euthanized by intraperitoneal injection of an overdose of sodium pentobarbital (150 mg/kg, >2× the anesthetic dose) in accordance with the American Veterinary Medical Association (AVMA) Guidelines for the Euthanasia of Animals (2020 Edition) (24).

### 2.2 FDM model construction

The FDM model was established using a modified face mask-based occlusion technique. A non-toxic, semi-transparent latex balloon (10 inches in diameter) was trimmed to fit the head of each guinea pig, ensuring complete coverage of the right eye while leaving the left eye, nostrils, mouth, and ears unobstructed (25). The occlusive mask was maintained continuously for four weeks. Daily inspections were conducted to verify mask integrity and to prevent mechanical compression or accidental visual exposure of the occluded eye.

### 2.3 Experimental groups and interventions

Recombinant human brain-derived neurotrophic factor (BDNF; Catalog No. P1007, APExBIO, USA) was used in this study. The lyophilized powder was reconstituted in sterile phosphate-buffered salin (PBS, pH 7.4), which served as the vehicle for BDNF administration, to a stock concentration of 0.2 mg/mL following the manufacturer’s instructions. Aliquots were stored at −20 °C until use. The primary outcome measure was refractive error and axial length. The sample size (n=9 per group) was selected based on prior studies in the guinea pig form-deprivation myopia model, which demonstrated that group sizes of 8–10 animals provide adequate statistical power to detect biologically relevant differences in myopic shift (26, 27). Initially, 45 tricolor guinea pigs were randomly allocated to five groups (n=9 per group) to determine the optimal BDNF concentration. The BDNF concentrations were selected based on previous administration studies (5). Group assignment was performed using a computer-generated random sequence (Microsoft Excel, RAND function). The groups included: FDM group(right eye occluded for four weeks without any intervention), FDM + Phosphate Buffered Saline (PBS) group (right eye occluded for four weeks, followed by a 2 μL intravitreal injection of PBS), FDM + 50 μg/mL BDNF group(right eye occluded for four weeks, followed by a 2 μL intravitreal injection of BDNF at a concentration of 50 μg/mL), FDM + 100 μg/mL BDNF group (right eye occluded for four weeks, followed by a 2 μL intravitreal injection of BDNF at a concentration of 100 μg/mL), FDM + 200 μg/mL BDNF group (right eye occluded for four weeks, followed by a 2 μL intravitreal injection of BDNF at a concentration of 200 μg/mL). After identifying 200 μg/mL as optimal, a second phase with 52 guinea pigs randomly divided into four groups (n = 13 per group) further studied BDNF effects on FDM progression. NC group (group A): normal rearing for four weeks without any intervention. NC + BDNF group (group B): normal rearing for four weeks, followed by a 2 μL intravitreal injection of BDNF at 200 μg/mL. FDM group (group C): right eye occluded for four weeks without any intervention. FDM+BDNF group (group D): right eye occluded for four weeks, followed by a 2 μL intravitreal injection of BDNF at 200 μg/mL. To minimize confounding, all outcome assessments (refraction, OCT, histology, and Western blot analysis) were conducted by investigators blinded to the experimental group. Additionally, the order of animal handling and measurements was randomized daily using a computer-generated sequence. Animals from different groups were housed in mixed cages or in cages randomly assigned across the holding rack to eliminate cage-location effects. All animals were marked on the dorsal surface with a non-toxic, animal-safe dye to ensure traceability throughout the experimental period. To minimize bias, experimental roles were separated among three researchers. Group allocation was performed by Researcher A, who also administered intravitreal injections. Tissue collection was carried out by Researcher B, and all outcome assessments (refraction, OCT, histology, Western blot) and data analyses were performed by Researcher C. Researchers B and C were blinded to group identities throughout the study; animals were identified only by coded labels. The allocation code was revealed only after completion of all analyses.

### 2.4 Intravitreal injection procedure

After covering the guinea pig’s right eye, administer a single intravitreal injection of the medication around 5:00 PM. Intravitreal injections were administered to the right eye after four weeks of occlusion. Prophylactic treatment with levofloxacin eye drops (Yabao Pharmaceutical Co., Ltd., China) was initiated one day before the procedure and applied three times daily to the right eye. Anesthesia was induced via intraperitoneal injection of 10% chloral hydrate (Shanghai Shanshu Chemical Co., Ltd., China) at a dosage of 0.25 mL/100 g. Cycloplegia was achieved using tropicamide eye drops (Santen Pharmaceutical Co., Ltd., Japan), and topical anesthesia of the corneal surface was provided using proparacaine hydrochloride eye drops (Alcon, USA).

The injection site was located approximately 1 mm posterior to the temporal limbus. A 32G microinjector was used to perform the injection under a surgical microscope at a 45° angle. Following the procedure, levofloxacin eye drops were continued three times daily to prevent postoperative infection. Animals in the FDM group underwent right-eye occlusion only, without intravitreal injection.

### 2.5 Biological measurements

Due to the high cumulative stress associated with the combination of form deprivation, repeated anesthesia, and intravitreal injections, we encountered significant mortality beyond 24 hours post-injection in our pilot studies. Therefore, in accordance with animal welfare guidelines (3R principles), the primary endpoint for the main experiment was set at 24 hours post-final injection to ensure sufficient survival for statistical analysis.

Biological parameters, including refractive error and axial length (AL), were assessed at three time points: prior to model induction, following four weeks of ocular occlusion, and one day after intravitreal injection.

#### 2.5.1 Refractive error measurement

Tropicamide eye drops (Santen Pharmaceutical Co., Ltd., Japan) were instilled into both eyes at 5-minute intervals for a total of four applications. After a 30-minute interval, streak retinoscopy (66 Vision-Tech Co., Ltd., China) was performed in a darkened room at a distance of 50 cm from the tested eye. The spherical equivalent was calculated as the mean of the horizontal and vertical refractive error values (28).

#### 2.5.2 Axial length measurement

AL was measured using the AVISO Ophthalmic A-Scan Ultrasound System (Quantel Medical, France). The ultrasound velocity settings were configured as follows: 1, 558 m/s for the anterior chamber, 1, 723 m/s for the lens, and 1, 540 m/s for the vitreous cavity. The gain was adjusted to 80–90 fdB. Proparacaine hydrochloride eye drops (Alcon, USA) were used for corneal surface anesthesia. Manual measurements were obtained in manual mode using indirect infiltration, with the probe positioned perpendicular to the corneal center. Ten measurements were obtained per eye, and the mean value accurate to 0.01 mm was recorded as the final AL (29).

### 2.6 Optical coherence tomography

OCT imaging was conducted on the experimental eyes of Group D at three time points: prior to the experiment, after four weeks of occlusion, and one day following intravitreal injection. The SARIS Small Animal Ophthalmic Multimodal Imaging Platform (Nanjing Boshi Medical Technology Co., Ltd., China) was used for image acquisition (n=3).

Anesthesia was induced with 5% pentobarbital sodium administered via intraperitoneal injection. Cycloplegia was achieved using tropicamide eye drops (Santen Pharmaceutical Co., Ltd., Japan), and topical corneal anesthesia was provided using proparacaine hydrochloride eye drops (Alcon, USA). Animals were positioned securely on the imaging platform, and carboxymethylcellulose gel was applied to the ocular surface to maintain corneal hydration during the procedure.

OCT scans were performed by a single experienced operator. Images were centered on the optic disc, and only those with clear resolutions were selected for analysis. Each eye was scanned three times, and the resulting images were recorded for subsequent evaluation.

### 2.7 Immunofluorescence staining and histopathological analysis

Three guinea pigs were randomly selected from the normal control group, and the expression of BDNF in their eyes was detected using immunofluorescence. Following euthanasia via administration of excess anesthetic, the eyes were promptly enucleated and fixed in 0.4% paraformaldehyde. Continuous paraffin sections (5 μm thickness) were prepared along the optic nerve axis. Sections were subjected to a standard protocol including dewaxing, hydration, antigen retrieval, permeabilization, and blocking (30). Subsequent incubation was performed with a primary antibody targeting brain-derived neurotrophic factor (BDNF, 1:100), followed by a secondary antibody (1:5000). Nuclear staining was conducted using 4′, 6-diamidino-2- phenylindole, after which the slides were mounted for microscopic observation.

### 2.8 Hematoxylin and eosinstaining

Paraffin sections underwent dewaxing, rehydration, hematoxylin staining, differentiation, eosin staining, dehydration, and clearing. After staining, sections were mounted for analysis (31).

For each group (n=4 per group), three paraffin sections containing cross-sections of the optic nerve were selected. Choroidal thickness (ChT) was measured using ImageJ software, with six repeated measurements taken per field of view. Due to the anticipated cumulative stress from repeated anesthesia and intravitreal injections, as well as the prolonged form deprivation period, animal survival was monitored throughout the experiment. Tissues from deceased animals were collected and preserved for potential ancillary analyses. However, to ensure temporal consistency and minimize confounding variables, only data from animals that survived the entire experimental protocol and were euthanized at the final time point were included in the primary statistical analysis (n = 4 per group). We conducted a total of three preliminary experiments, retaining a portion of tissue from each. All tissue samples were re-examined, and the choroidal thickness was calculated for each group of guinea pig samples (n=4).

### 2.9 Western blot analysis

Following the final measurements, euthanasia was performed, and retinal tissues were carefully dissected for protein extraction. Retinas were lysed in a buffer containing protease inhibitors, homogenized, and sonicated. The lysates were centrifuged at 12, 000 r/min for 15 minutes at 4 °C, and the resulting supernatants were collected. Protein concentrations were determined using the bicinchoninic acid method. Samples were denatured by heating with loading buffer at 100 °C for 10 minutes.

Proteins were separated by 10% sodium dodecyl sulfate-polyacrylamide gel electrophoresis and subsequently transferred onto polyvinylidene difluoride membranes. Membranes were blocked with 5% bovine serum albumin and incubated overnight at 4 °C with primary antibodies targeting BDNF (at 1:1000, HA722912, HuaBio, China), PI3K (at 1:1000, T40115, Abmart, China), AKT (at 1:1000, T55561, Abmart, China), eNOS (at 1:1000, 27120-1-AP, Proteintech, China), and nNOS(at 1:1000, ET1609-61, HuaBio, China). After washing with tris-buffered saline containing Tween-20, membranes were incubated with horseradish peroxidase-conjugated secondary antibodies (1:5000) at room temperature.

Detection was conducted using enhanced chemiluminescence (Thermo Fisher Scientific, USA), and the intensities of protein bands were quantified using ImageJ software. All values were normalized to glyceraldehyde-3-phosphate dehydrogenase (GAPDH) (at 1:10000, 10494-1-AP, Proteintech, China) expression. Data represent the results of three independent biological experiments, 3–5 guinea pigs per group were used. In total, 9-12 independent retinal samples were analyzed per group. Bar graphs depict the statistical analysis of these three independent experimental outcomes to ensure reproducibility and reliability.

### 2.10 Statistical analysis

All animals that entered the study were included in the final analysis; there were no exclusions at the animal or data-point level. All data were analyzed using GraphPad Prism version 8.0.1. Normality was assessed by the Shapiro–Wilk test, and homogeneity of variance by Brown–Forsythe’s test. All outcome measures met the assumptions for parametric testing; therefore, paired t-tests (within-group eye comparisons), one-way ANOVA (between-group comparisons), and two-way repeated measures ANOVA (longitudinal analysis) were used, followed by Tukey’s multiple comparisons test for pairwise comparisons. Results are expressed as mean ± SD. p < 0.05 was considered statistically significant.

## 3 Results

### 3.1 Effects of BDNF on refractive error and axial length in guinea pigs

Data on refractive error and AL for both eyes across the five experimental groups at various time points are presented in **Table 1**. Before the experimental intervention, all groups exhibited a hyperopic refractive status, with mean refractive errors of approximately +5.00 D. After four weeks of monocular occlusion, experimental eyes in all groups demonstrated a myopic shift accompanied by axial elongation. Compared with the contralateral control eyes, significantly increased myopic refractive error and AL were observed in the experimental eyes (*p* < 0.01), confirming the successful establishment of FDM. No statistically significant differences in refractive error or AL were found between the experimental and control eyes either before or immediately after occlusion (*p* > 0.05), indicating consistent baseline characteristics across groups.

**Table 1:** Effects of different BDNF concentrations on refractive error and axial length in guinea pigs.

Groups		Pre-treatment		Post-occlusion		Post-injection	
		experimental eyes	control eyes	experimental eyes	control eyes	experimental eyes	control eyes
Refractive Error (D)							
FDM		4.59±1.08	4.72+1.53	1.48±1.07**	4.17±1.41	1.4±1.17**	4.17±1.33
FDM+PBS		5.06±1.07	5.32±1.32	1.26±0.87**	4.58±1.40	1.19±1.09**	4.61±1.42
FDM+BDNF	50μg/ml	5.17±0.86	5.47±1.39	1.16±1.14**	4.64±1.29	2.21±1.18**	4.56±1.22
	100μg/ml	5.48±0.91	5.69±0.73	2.1±1.05**	4.94±0.79	3.18±1.01**	4.89±0.73
	200μg/ml	5.09±0.74	5.17±1.58	1.19±0.88**	4.42±1.67	3.98±0.98	4.39±1.66
Axial Length (mm)							
FDM		7.94±0.14	7.94±0.12	8.52±0.08**	8.28±0.10	8.53±0.09**	8.28±0.10
FDM+PBS		7.92±0.09	7.88±0.07	8.53±0.01**	8.34±0.11	8.53±0.08**	8.33±0.10
FDM+BDNF	50μg/ml	7.99±0.1	7.96±0.11	8.58±0.06**	8.35±0.09	8.49±0.1*	8.36±0.09
	100μg/ml	7.88±0.11	7.88±0.07	8.53±0.12**	8.30±0.08	8.43±0.13*	8.30±0.08
	200ug/ml	7.91±0.12	7.89±0.11	8.58±0.11**	8.32±0.14	8.38±0.11	8.34±0.13

*p < 0.05, **p < 0.01 compared to the control eye in the same group at the same time point.

On the first day following intravitreal injection of various BDNF concentrations, one-way ANOVA indicated statistically significant differences among the experimental eyes of the groups in both refractive error and AL (*p* < 0.05). Pairwise comparisons showed that the refractive error in the FDM + 200 μg/mL BDNF group was significantly more hyperopic and the AL significantly shorter, compared with the FDM and FDM + PBS groups (refractive error: *p* < 0.01; AL: *p* < 0.05). Additionally, a significantly lower myopic refractive error was observed in the FDM + 100 μg/mL BDNF group compared with the FDM + PBS group (*p* < 0.05).

**Figure 1:**
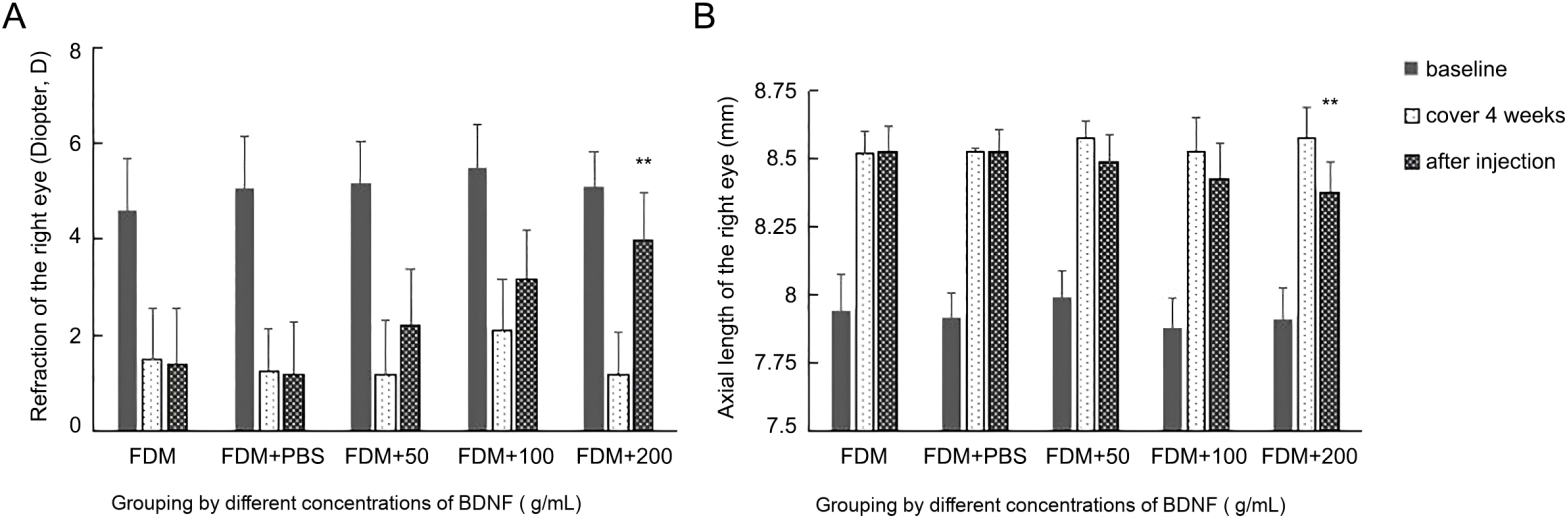
Dose-effect relationship of BDNF on refractive error and axial length in FDM guinea pigs. **(A)** Effect of BDNF on refractive error in FDM guinea pigs. **(B)** Effect of BDNF on axial length in FDM guinea pigs. Data are presented as mean ± SD. **p < 0.001 versus FDM and FDM+PBS groups (n = 9 per group).

Two-way repeated measures ANOVA was used to assess changes in refractive error and AL across three time points (baseline, after four weeks of occlusion, and one day post-injection). No significant changes in refractive error or AL were observed in the FDM, FDM + PBS, FDM + 50 μg/mL BDNF, and FDM + 100 μg/mL BDNF groups between the pre- and post-injection measurements (*p* > 0.05). In contrast, the FDM + 200 μg/mL BDNF group indicated statistically significant differences in both refractive error and AL before and after injection (*p* < 0.001) (**Figure 1**), indicating that intravitreal administration of BDNF at 200 μg/mL effectively inhibited myopia progression in the FDM model.

**Table 2** shows the refractive power and axial length of the four groups of guinea pigs at different time points in Experiment 2. The results indicate that intravitreal injection of a 200 μg/ml BDNF solution had no effect on the axial length or refractive power of normal guinea pigs, but it did inhibit the axial length and myopic refractive power in FDM guinea pigs.

**Table 2:** Effects of BDNF on eye axis length and refractive power in guinea pigs.

Groups	Parameter	Pre-treatment		Post-occlusion		Post-injection	
		experimental eyes	control eyes	experimental eyes	control eyes	experimental eyes	control eyes
A	Refractive Error (D)	4.14±1.39	4.72±1.18	3.67±1.31	3.78±0.79	3.50±1.27	3.78±0.80
B		4.64±1.71	4.75±1.63	4.81±1.26	4.42±1.62	4.75±1.25	4.28±1.70
C		4.14±1.39	4.72±1.18	-0.08±2.36*	3.78±0.79	-0.08±2.33*	3.78±0.80
D		4.61±1.52	4.94±1.07	-0.25±1.58*	3.92±1.66	3.39±1.60	3.94±1.84
A	Axial Length (mm)	7.97±0.17	7.94±0.14	8.37±0.11	8.33±0.14	8.38±0.12	8.31±0.15
B		7.99±0.17	7.89±0.13	8.26±0.08	8.24±0.10	8.25±0.07	8.23±0.11
C		7.97±0.17	7.94±0.14	8.57±0.11*	8.33±0.14	8.56±0.13*	8.30±0.15
D		7.95±0.08	7.91±0.13	8.58±0.13*	8.32±0.11	8.37±0.09	8.30±0.11

Group A: No treatment to either eye; Group B: After 4 weeks of normal rearing, 2 μl of BDNF solution (200 μg/ml) was injected into the vitreous cavity of the right eye; Group C: The right eye was occluded for 4 weeks; Group D: After 4 weeks of occlusion of the right eye, 2 μl of BDNF solution (200 μg/ml) was injected into the vitreous cavity of the right eye. *p < 0.05, compared to the control eye in the same group at the same time point., n=9.

### 3.2 Immunofluorescence localization of BDNF

**Figure 2:**
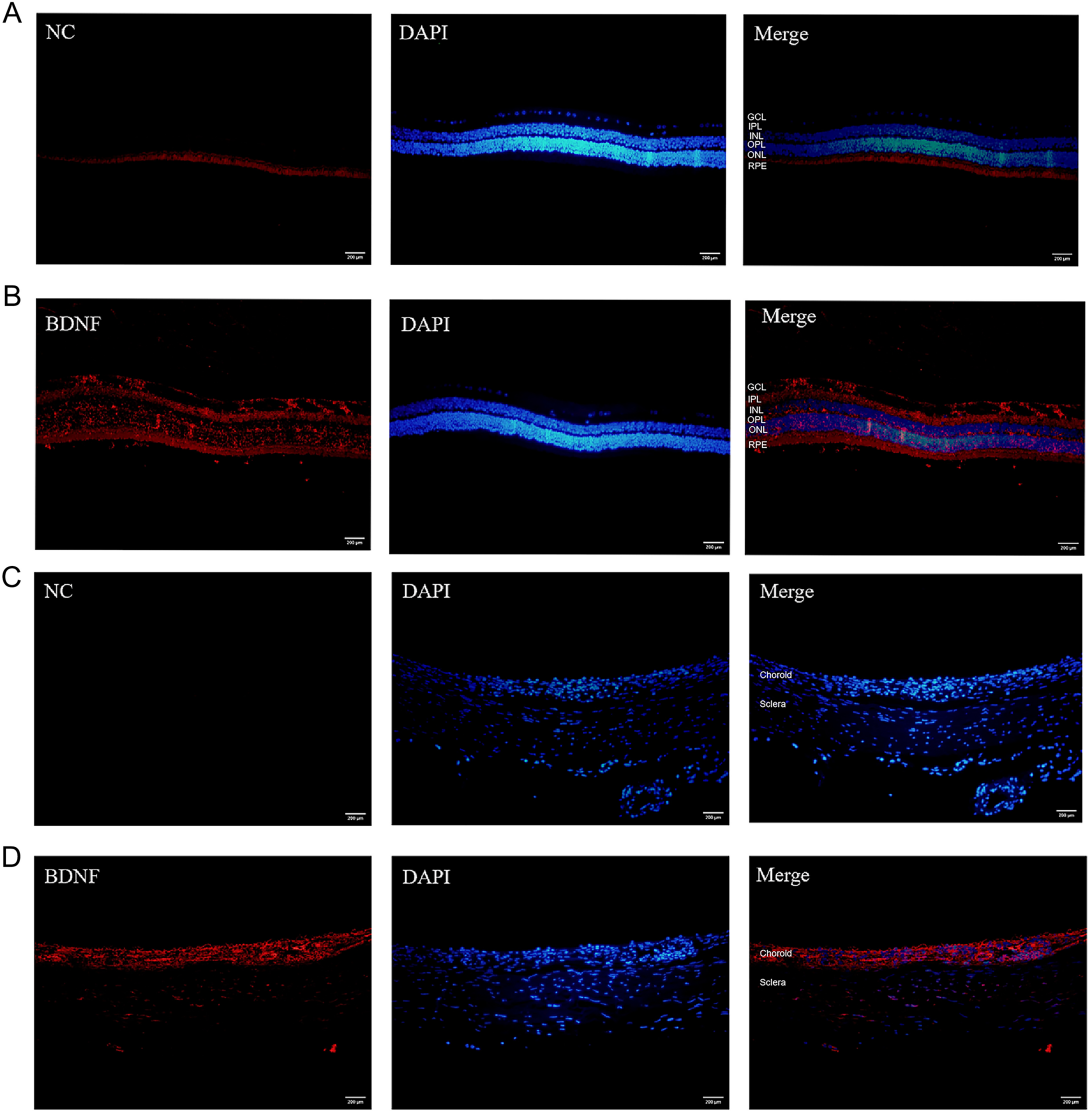
Immunofluorescence localization of BDNF in guinea pig retina and choroid. **(A)** BDNF expression in the NC group (retina). **(B)** BDNF expression in the BDNF group (retina). **(C)** BDNF expression in the NC group (choroid and sclera). **(D)** BDNF expression in the BDNF group (choroid and sclera). Scale bar = 200 μm. (n = 3 per group).

Immunofluorescence analysis indicated strong fluorescence signals throughout the retinal tissue, indicating widespread distribution of BDNF in the guinea pig retina. BDNF expression was predominantly localized to the retinal ganglion cell layer, inner nuclear layer, and outer plexiform layer. High fluorescence intensity was also observed in the choroid, while only weak signals were present in the scleral tissue, suggesting greater BDNF abundance in the choroid compared with the sclera (**Figure 2**).

### 3.3 Histopathological analysis

**Figure 3:**
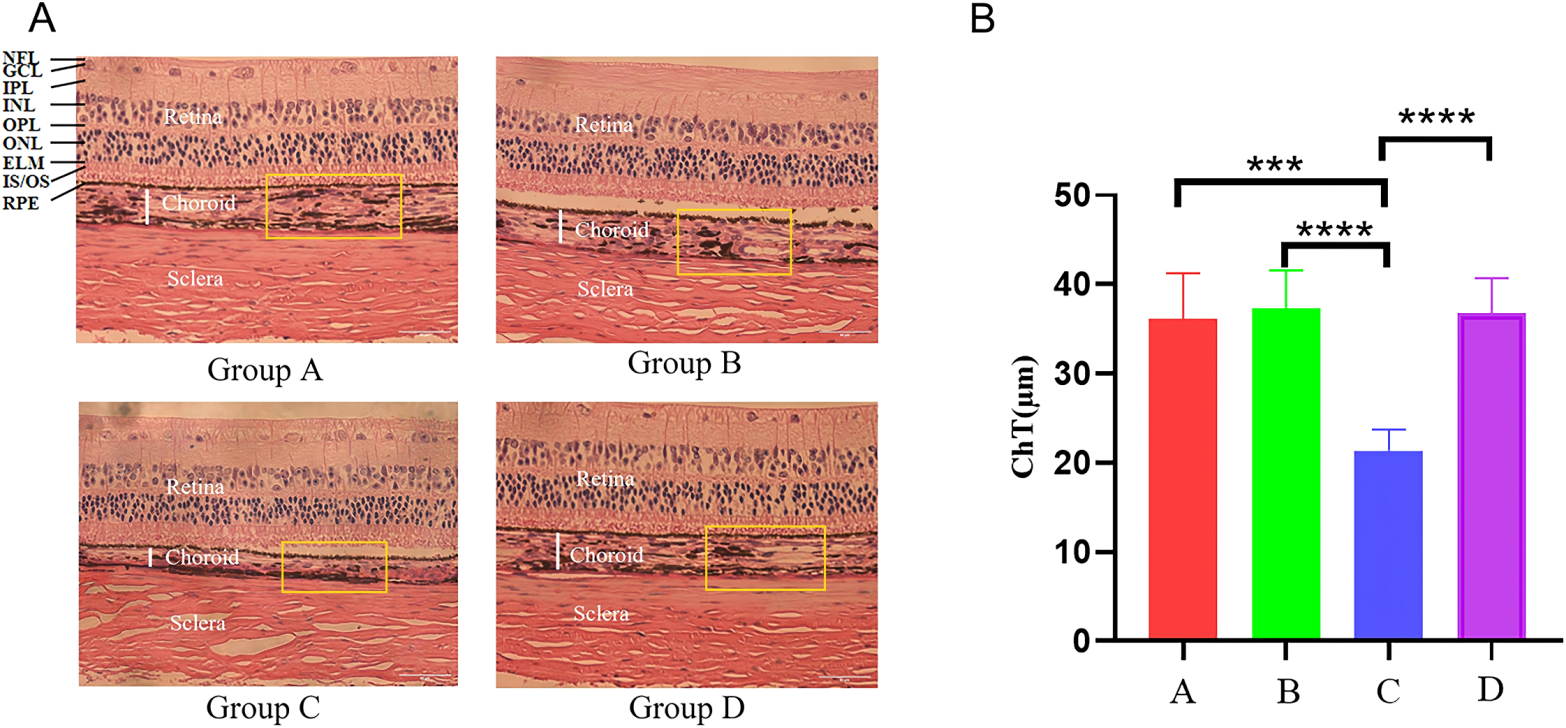
Histopathological changes in choroidal and scleral tissues. **(A)** Morphological changes in four groups (400×, scale bar = 50 μm). **(B)** Comparative choroidal thickness among groups (x̄ ± s, μm). ***p < 0.001, ****p < 0.0001. Groups: Group A (NC): No intervention; Group B (NC+BDNF): Intravitreal BDNF injection after 4 weeks of normal rearing; Group C (FDM): 4 weeks of occlusion; Group D (FDM+BDNF): Intravitreal BDNF injection after 4 weeks of occlusion. (n=4 per group).

H&E staining revealed that choroidal thickness (ChT) in the FDM group was significantly reduced compared with both the NC and NC + BDNF groups (*p* < 0.0001), indicating that form deprivation induced choroidal thinning in the guinea pig model. In contrast, intravitreal BDNF administration (FDM + BDNF group) significantly increased ChT when compared with the FDM group (*p* < 0.0001), demonstrating the ability of BDNF to mitigate form-deprivation-induced choroidal thinning. Additionally, BDNF treatment improved the disorganization of collagen fibers and increased inter-fibrillar spacing in the sclera, which had been disrupted by form deprivation (**Figure 3**).

### 3.4 OCT findings

**Figure 4:**
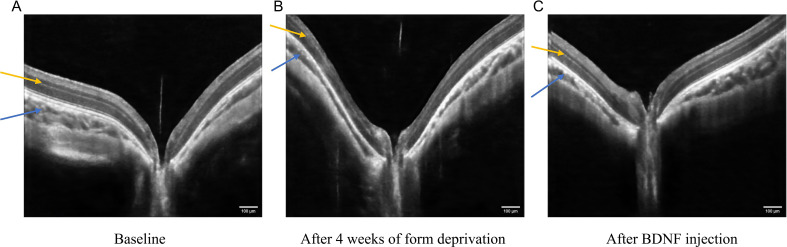
Changes in retinal and choroidal thickness assessed by OCT during form-deprivation myopia and after BDNF intervention. Yellow arrows indicate the retina, and blue arrows indicate the choroid. The images show representative cross-sectional OCT scans at **(A)** baseline, **(B)** after 4 weeks of form deprivation, and **(C)** after intravitreal BDNF injection (n = 3 per group).

OCT imaging showed that, compared with baseline measurements, both retinal and ChT in the experimental eyes were significantly reduced after four weeks of occlusion. However, following intravitreal BDNF injection, both retinal and choroidal thicknesses increased significantly compared with post-occlusion values (**Figure 4**).

### 3.5 Retinal protein expression changes

Western blot analysis showed that retinal BDNF protein expression in the FDM group was significantly lower than that in the NC group (*p* < 0.05), indicating monocular occlusion reduced BDNF expression in the guinea pig retina. In contrast, BDNF expression was significantly higher in the NC+BDNF group than in the NC group. (*p* < 0.05), and the FDM + BDNF group also increased relative to the FDM group. (*p* < 0.01), indicating the intravitreal BDNF administration significantly increased total retinal BDNF protein expression, supporting effective delivery to the retina.

No significant differences were observed in the expression levels of PI3K, AKT, or eNOS proteins between the NC and NC + BDNF groups (*p* > 0.05). However, nNOS protein expression was significantly upregulated in the NC + BDNF group compared with the NC group (*p* < 0.01), indicating that intravitreal administration of BDNF may selectively influence nNOS expression without significantly affecting PI3K, AKT, or eNOS levels.

**Figure 5:**
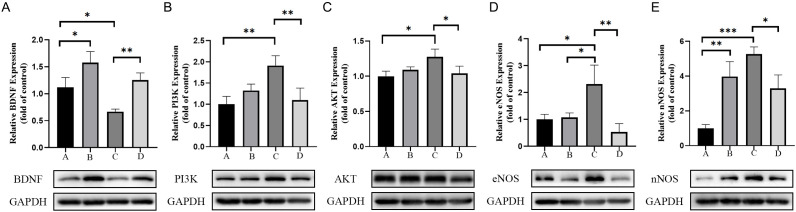
Retinal protein expression profiles. **(A–E)** Protein expression and grayscale analysis in experimental eyes after injection. Groups: Group A (NC), Group B (NC+BDNF), Group C (FDM), Group D (FDM+BDNF). *p < 0.05, **p < 0.01, ***p < 0.001. Total 9–12 independent retinal samples per group, derived from three independent experimental runs.

Compared with the NC group, the FDM group showed a significant increase in the protein expression of PI3K, AKT, eNOS, and nNOS in guinea pig retinas, with all differences being statistically significant (*p* < 0.05), indicating that visual deprivation leads to elevated levels of PI3K, AKT, eNOS, and nNOS proteins in the retinas of normal guinea pigs. In contrast, the expression levels of PI3K, AKT, eNOS, and nNOS proteins in the retinas of guinea pigs in the FDM+BDNF group were lower than those in the FDM group, with statistically significant differences (*p* < 0.05); this suggests that intravitreal injection of BDNF solution can downregulate the expression levels of PI3K, AKT, eNOS, and nNOS proteins in the retinas of FDM guinea pigs (**Figure 5**).

## 4 Discussion

Myopia is a major public health concern worldwide, and BDNF has emerged as a potential regulator of ocular growth and myopia progression. Within retinal tissues, BDNF is primarily synthesized by retinal glial cells, retinal ganglion cells, and amacrine cells, and is delivered to target sites through paracrine and autocrine mechanisms (14, 15). By binding to tropomyosin receptor kinase B (TrkB), BDNF plays a key role in neuronal survival, trophic support, morphological development, and neuroprotection following injury (32). The protective role of BDNF in the retina and optic nerve has been well documented (14, 15). Notably, BDNF levels are significantly reduced in the aqueous humor of patients with high myopia, supporting its involvement in myopic pathogenesis (16). Previous studies have proposed BDNF as a potential therapeutic target for myopia control (17).

The aim of the present study was to investigate the effect of intravitreal BDNF injection on ocular growth, retinal and choroidal structure, and related signaling pathways in a guinea pig model of form−deprivation myopia. In the present study, intravitreal BDNF injection did not alter axial length or refractive status in normally developing guinea pigs, suggesting that BDNF does not interfere with physiological ocular growth. As a large molecular protein of the neurotrophin family, BDNF has a relatively short *in vivo* half-life (32). In glaucoma models, BDNF delays or prevents the degeneration of RGCs, although its neuroprotective effects are time-limited (33). Similarly, in optic nerve injury models, exogenous BDNF supplementation has demonstrated beneficial but transient effects (34, 35). Based on these findings, ocular biometric parameters were measured one day following intravitreal BDNF administration in the present study. Our results showed that intravitreal injection of 200 μg/mL BDNF significantly increased refractive diopter and reduced AL in FDM guinea pigs, indicating that BDNF may delay the progression of FDM.

Given that retinal structure is critical for maintaining normal ocular growth, we further assessed retinal thickness using OCT to clarify the structural changes associated with BDNF’s anti-myopic effect. OCT enables non-invasive, high-resolution, and repeated *in vivo* imaging of the retina, retinal pigment epithelium, and choroid (36). To reduce potential deviations introduced by retinal detachment during histopathological analysis, OCT was used in this study to monitor retinal thickness in guinea pigs. Our results demonstrated that form deprivation resulted in a decrease in retinal thickness, which are consistent with previous studies in rodent models (37), whereas intravitreal BDNF administration promoted partial restoration of retinal thickness in FDM guinea pigs.

The choroid, a vascularized component of the uveal tract situated between Bruch’s membrane and the sclera, extends from the ora serrata to the optic nerve (36). Numerous studies have emphasized the key role of choroids in ocular growth regulation and the pathogenesis of myopia. Clinical studies have consistently demonstrated that ChT is significantly reduced in myopic eyes when compared with emmetropic and hyperopic counterparts (38–41). Choroidal thinning has been recognized as a characteristic anatomical change associated with myopia, and ChT shows a negative correlation with AL, a relationship closely linked to both the development and progression of myopia (42, 43). Given this correlation, changes in ChT have emerged as potential indicators for monitoring myopia progression and evaluating the efficacy of myopia control interventions (44).

Form deprivation disrupts the formation of clear retinal images, transmitting persistent blurred visual signals through the choroid to the sclera. This process contributes to extracellular matrix remodeling in the sclera, subsequently promoting ocular elongation and inducing thinning of both the sclera and choroid (45, 46). Recent domestic and international studies have further confirmed that form deprivation leads to significant scleral and choroidal thinning in guinea pigs (47–49). In this study, H&E staining revealed that four weeks of form deprivation resulted in sparse scleral architecture, disorganized collagen fibers, increased inter-fibrillar spacing, and choroidal thinning in the experimental eyes. Intravitreal BDNF administration mitigated both choroidal thinning and scleral disorganization. Additionally, OCT results showed that ChT increased following BDNF administration in FDM guinea pigs, suggesting that enhanced ChT may contribute to the prevention or delay of myopia onset (47).

In this study, immunofluorescence techniques were used to determine the localization of BDNF in guinea pig ocular tissues. BDNF was primarily distributed in the retina and choroid, with expression observed across multiple retinal layers, including the retinal ganglion cell layer, inner nuclear layer, and outer plexiform layer. Furthermore, immunohistochemical analysis confirmed the presence of BDNF and its receptor, TrkB, in the retinal ganglion cell layer, outer plexiform layer, inner nuclear layer, and optic disc region (50). Based on these findings, retinal tissue was subsequently extracted for quantitative analysis of BDNF and related protein expression levels.

The PI3K/AKT signaling pathway, consisting of core components PI3K and AKT, is a key regulatory mechanism in mammalian cells and is widely expressed across diverse cell types (19). This pathway is activated by various intrinsic and extrinsic stimuli and modulates the activity of multiple downstream effector proteins, thereby influencing key cellular processes including growth, proliferation, differentiation, and metabolism (51). Increasing evidence indicates that the PI3K/AKT pathway plays a significant role in the onset and progression of myopia. In lens-induced myopia models, activation of the PI3K/AKT/ERK signaling cascade has been associated with enhanced retinal fibrosis, leading to retinal thinning and functional impairment that may exacerbate myopic progression (20). Consistent with these reports, our study found that visual deprivation in form-deprived myopia models leads to elevated expression levels of PI3K, AKT, and their downstream effectors eNOS and nNOS in the retinas of guinea pigs.

In normally developing guinea pigs, intravitreal BDNF injection did not significantly alter PI3K or AKT expression levels in the retina, indicating minimal impact on this pathway under physiological conditions. Following successful induction of FDM through four weeks of monocular occlusion, PI3K and AKT protein expression levels in the retina were significantly elevated. However, after BDNF injection, a significant increase in refractive diopter, reduction in AL, and downregulation of PI3K and AKT expression were observed. These findings indicate that BDNF may attenuate myopic progression by modulating the PI3K/AKT signaling pathway.

NO has been identified as a key signaling molecule within the PI3K/AKT pathway. Under hypoxic conditions, activation of PI3K/AKT leads to phosphorylation of eNOS (52). Upregulation of this pathway has also been implicated in the pathophysiology of myopia (20), and inhibition of NOS suppresses ChT induced by myopic defocus (53). These findings reinforce the central role of NO in the PI3K/AKT signaling cascade. As a key mediator of oxidative stress responses, NO may contribute to myopia progression through modulation of related signaling pathways (21).

NO is synthesized from L-arginine by NOS enzymes, which catalyze its production as an endothelium-derived relaxing factor (54). Three NOS isoforms are recognized: nNOS, iNOS, and eNOS (22). Both nNOS and eNOS are constitutively expressed, whereas iNOS is typically absent under physiological conditions but can be induced in response to immune stimuli (55). In humans, nNOS is primarily expressed in the nervous system, facilitating neural signal transmission; iNOS is upregulated during inflammation or immune activation and produces large quantities of NO for immune defense; and eNOS is mainly localized to vascular endothelial cells, contributing to vasodilation (56). Due to the absence of significant inflammation in our form−deprivation myopia model, iNOS protein expression was negligible (56). Therefore, we focused our analysis on eNOS and nNOS.

Prior studies have demonstrated that NO plays a significant regulatory role in the retina during myopia development (23, 57). In related research, BDNF has been reported to exert partial effects in FDM through modulation of nNOS expression (16). For example, Yu et al. reported elevated mRNA and protein levels of eNOS and nNOS in the choroid of myopic guinea pigs, indicating that NO may help regulate ChT and blood flow during myopic progression (58). Other studies have reported increased retinal iNOS expression in FDM models, with NOS inhibitors effectively suppressing the onset of myopia (59). Previous research has also reported upregulated expression of eNOS and nNOS in lens-induced myopia models, where intravitreal NOS inhibitor injection reduced activation of NO-related signaling molecules and preserved ChT (60).

As eNOS is primarily involved in vascular dilation and no vascular changes were observed in healthy guinea pigs, intravitreal BDNF administration did not significantly influence eNOS expression in these animals (56). However, an upregulation of nNOS expression was observed in normal guinea pig retinas following BDNF injection, possibly attributable to the role of nNOS in synaptic plasticity and neuronal function (61). BDNF may support retinal neuron survival and function through binding to its receptor TrkB and activation of downstream signaling pathways (61). Consequently, elevated BDNF levels may indirectly enhance nNOS expression via neuroprotective mechanisms. Thus, the translational potential of BDNF-based neuroprotection is supported by recent evidence in ocular pharmacology. Bucolo et al. demonstrated the feasibility of pharmacological interventions in eye disease models (62). Godos et al. highlighted neuroprotective strategies relevant to ocular health (63), while Amato et al. emphasized the critical role of neurotrophic support in retinal ganglion cell survival (64). Together, these findings suggest that BDNF-mediated neuroprotection, as demonstrated in our study, may represent a viable translational approach for myopia control.

In this study, increased eNOS and nNOS expression was observed in the retinas of FDM guinea pigs. This elevation was significantly attenuated following intravitreal BDNF administration, indicating a regulatory role of BDNF in myopia progression through modulation of eNOS and nNOS expression.

In addition to the intravitreal BDNF injection used in the present study, which is effective but invasive and limited by the short half−life of exogenous BDNF, an alternative promising strategy is to upregulate endogenous retinal BDNF expression via topical application of small neuroprotective molecules. For example, brimonidine, an α2−adrenergic agonist widely used as an eye drop for glaucoma, has been shown to significantly increase retinal BDNF levels and protect retinal ganglion cells in animal models of retinal injury (65). Similarly, caffeine, a well−tolerated psychoactive compound, can enhance BDNF expression and exert neuroprotective and anti−inflammatory effects in the retina (66). These findings suggest that topical delivery of such non−invasive, clinically available agents may represent a feasible and translational approach to sustainably elevate retinal BDNF and modulate the BDNF/TrkB pathway, thereby delaying myopia progression without the limitations of repeated intraocular injections.

Taken together, the findings suggest that BDNF may exert beneficial effects in the FDM guinea pig model through multiple mechanisms: downregulation of PI3K, AKT, eNOS, and nNOS expression in the retina; enhancement of retinal and ChT; and reduction of scleral disorganization and collagen sparsity. These results support BDNF as a promising therapeutic target for myopia control rather than a direct clinical treatment, and provide experimental evidence for the development of future anti−myopia strategies.

### 4.1 Limitations and prospects

A major limitation of this study is the single 24-hour endpoint, required due to high pilot mortality during longer follow-up from invasive procedures, meaning our findings reflect acute rather than long-term pharmacodynamic effects. Future studies using less invasive delivery (e.g., topical administration) are needed to assess durable myopia control via sustained BDNF signaling. While we observed an association between BDNF treatment, PI3K/AKT/nNOS modulation, and myopia control, causality cannot be confirmed without pathway-specific inhibitor studies. Additionally, concurrent molecular and biometric measurements at 24 hours do not establish temporal precedence, requiring future time-course studies. Finally, sex-specific analyses were not performed due to small sample size; future large-sample studies should explore potential sex-dependent anti-myopic effects of BDNF, given evidence that sex influences ocular growth regulation (67–69).

## Data Availability

The original contributions presented in the study are included in the article/supplementary material. Further inquiries can be directed to the corresponding authors.
